# Identification of potential molecular pathogenesis mechanisms modulated by microRNAs in patients with Intestinal Neuronal Dysplasia type B

**DOI:** 10.1038/s41598-019-54245-4

**Published:** 2019-11-27

**Authors:** Marcos C. Angelini, Alana Maia e. Silva, Tainara F. Felix, Rainer M. L. Lapa, Simone A. Terra, Maria A. M. Rodrigues, Erika V. P. Ortolan, Patricia P. Reis, Pedro L. T. A. Lourenção

**Affiliations:** 1 UNESP - São Paulo State University, Faculty of Medicine, Department of Surgery and Orthopedics, Botucatu, SP Brazil; 2 UNESP - São Paulo State University, Faculty of Medicine, Experimental Research Unity (UNIPEX), Botucatu, SP Brazil; 3 Institute of Livestock and Biotechnology, Laboratory of Molecular Physiology, Toribio Rodriguez de Mendoza National University, Amazonas, Peru; 4 UNESP - São Paulo State University, Faculty of Medicine, Department of Pathology, Botucatu, SP Brazil

**Keywords:** Enteric neuropathies, Molecular medicine

## Abstract

This study proposed to determine global microRNA (miRNA) expression and miRNA-regulated pathways in Intestinal Neuronal Dysplasia type B (IND-B). Fifty patients (0–15 years old) with IND-B were included in the study. Peripheral blood samples were collected from all 50 patients and from 10 healthy asymptomatic children (controls). Rectal biopsies were collected from 29/50 patients; biopsy tissues were needle microdissected to isolate the different intestinal layers, for molecular analysis. Global miRNA expression was determined using TaqMan arrays. Correlation analysis between miRNA expression in plasma and biopsy samples as well as among tissues derived from the distinct intestinal layers was performed. Computational approaches were used for miRNA target prediction/identification of miRNA-regulated genes and enriched pathways biologically relevant to IND-B pathogenesis. miRNAs were statistically significantly deregulated (FC ≥ 2 and p ≤ 0.05) in submucosal and muscular layers: over-expressed (miR-146a and miR-146b) and under-expressed (miR-99a, miR-100, miR-130a, miR-133b, miR-145, miR-365, miR-374-5p, miR-451). Notably, let-7a-5p was highly over-expressed in patient plasma compared to healthy controls (FC = 17.4). In addition, miR-451 was significantly under-expressed in both plasma and all biopsy tissues from the same patients. Enriched pathways (*p* < 0.01) were axon guidance, nerve growth factor signalling, NCAM signalling for neurite out-growth, neuronal system and apoptosis. miRNA expression is deregulated in the submucosa and muscular layers of the rectum and detected in plasma from patients with IND-B. Biologically enriched pathways regulated by the identified miRNAs may play a role in IND-B disease pathogenesis, due to the activity related to the neurons of the enteric nervous system.

## Introduction

Intestinal neuronal dysplasia type B (IND-B) is a pathological entity of the group of gastrointestinal neuromuscular diseases characterized by complex alterations in the intestinal submucosal nerve plexuses^1^. Patients typically present with severe intestinal constipation, sometimes complicated by episodes of intestinal obstruction^2^. IND-B diagnosis relies on the histopathological analysis of rectal biopsies by identifying the hyperplasia of the submucosal nerve plexus^1,3^, which is characterized according to different criteria^1,4–6^. Despite the intense scientific research that has been performed in recent decades, IND-B still remains with uncertainties regarding its definition, etiopathogenesis, diagnostic criteria and treatment^2,7,8^.

IND-B etiopathogenesis is widely debated. There are three main theories that attempt to explain IND-B development. IND-B can be understood as a primary disease in which genetic alterations directly influence the embryological development of the enteric nervous system^2,9^. This theory is supported by *in vivo* studies demonstrating that certain mutations may play a role on migration and development of neural crest cells, determining megacolon and hyperplasia of neuroenteric plexuses^10,11^. However, these findings remain to be identified in human studies. Another theory states that IND-B should be understood as a secondary phenomenon due to congenital intestinal obstructions or local inflammatory processes^12,13^. Others believe that the morphological changes found in IND-B represent only one step in the development of the enteric nervous system and should be understood as a physiological maturation process^4,14,15^.

The discovery of regulatory small non-coding RNAs, in 1993^16^, has opened extensive avenues of research in many disease conditions. microRNAs (miRNAs) are a large class of conserved, small, noncoding RNAs of approximately 19-25 nucleotides length, which act in post-transcriptional regulation of target messenger RNA (mRNA). miRNAs have an important role in organ development and modulate mechanisms of cell differentiation, proliferation, migration, apoptosis, among other^17^. The growing knowledge about the role of miRNAs in disease development and progression have led to the establishment of miRNAs as potential biomarkers, clinically applicable for diagnosis, prognosis and therapeutic molecules^16,18–21^.

Therefore, we aimed to determine global miRNA expression profiles in microdissected biopsy tissues and plasma from patients diagnosed with IND-B. Our results are novel since we show deregulated expression of a subset of miRNAs in the submucosa and muscular layers of the rectum and in plasma from patients with IND-B. In addition, we identified biologically enriched pathways containing a large number of miRNA-target genes with roles related to the neurons of the enteric nervous system. Such pathways may play a role in IND-B disease pathogenesis.

## Methods

### Patients, healthy controls and study design

This study was approved by our local Research Ethics Board, Botucatu Medical School, UNESP, São Paulo, Brazil (REB#11520712.6.0000.541) and performed in accordance with national and international ethical guidelines and recommendations of the Declaration of Helsinki. Patients and/or their guardians were previously informed about the research, and invited to participate. Patients and guardians who accepted to participate in the study signed their respective informed consent.

**Figure 1:**
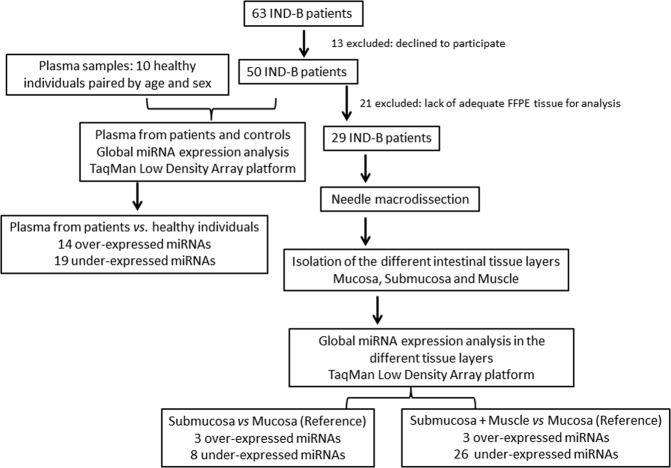
Flowchart integrating study design and main study results.

Figure 1 shows the study design including patients and methods of data analysis.

This is a single-center, translational study, which enrolled 63 patients (<15 years) diagnosed with IND-B at the University Hospital of the Botucatu Medical School, UNESP, between 1998 and 2012. The diagnosis of IND-B has been previously established based on histopathological analysis of rectal biopsies or surgical specimens according to the morphological criteria proposed by the Frankfurt Consensus, 1990, with no additional associated intestinal dysganglionosis^22^. Thirteen patients declined to participate and were therefore excluded from the study. Fifty patients were included in the study. In addition, 10 volunteers (<15 years) considered healthy and without gastrointestinal symptoms were invited to participate (reference control group) and provided a peripheral blood sample.

Global microRNA expression profiles were determined in plasma and rectal biopsy tissues. Analysis of plasma miRNA profiles were determined in 50 IND-B patients compared to the 10 healthy controls. Of these 50 patients, 29 had archived formalin-fixed, paraffin-embedded (FFPE) rectal tissue samples of adequate quantity and quality for microdissection and RNA extraction. Rectal tissue samples were pooled according to their location in the distinct layers of the intestinal wall. miRNA expression in intestinal submucosa and submucosa plus muscular layers was compared to the mucosal layer, since ganglion cells of the nerve plexus, which are affected in IND-B, are not present in this layer. By using this strategy, we were able to show deregulated miRNA expression in the intestinal layers containing ganglion cells of the nerve plexus, which are implicated in IND-B pathogenesis.

### Plasma samples

Peripheral blood samples (4 mL) were collected in Vacutainer® BD tube (Becton Dickinson, Franklin Lakes, NJ, USA) with EDTA and immediately processed to obtain cell-free plasma, by a two-step centrifugation at 1,200 rpm for 15 minutes at 4 °C. The plasma fraction (upper phase) was then transferred to a sterile, RNAse-free 1.5 mL eppendorf tube and stored at −80 °C until use.

### Needle microdissection of FFPE rectal biopsy tissues

We used 5 sections of 10 μm each for microdissection and RNA extraction, as follows: for tissue microdissection, slides were incubated in 100% Xylol (Merck, Darmstadt, Germany) for 10 minutes and then dehydrated and rehydrated in a series of ethanol: 100% for 5 minutes, 95% for 5 minutes, 80% for 5 minutes, 70% for 5 minutes. Tissues were subsequently stained in a solution of hematoxylin (5:1 dilution) for 15 s. For identification of microdissection areas, slides were stored in MilliQ ddH2O until the time of scraping (maximum 5 min.) with sterile hypodermic needle1.60 × 40 mm (Becton Dickinson, Curitiba, PR, Brazil). The areas corresponding to each of the layers of the intestinal wall were dissected using the Leica EZ4 stereomicroscope (Leica Microsystems, China) for visualization and scraping of the tissues. Microdissected samples were pooled according to the intestinal wall layers: mucosa, submucosa and muscular layer. Each of the pooled samples was placed in a sterile, RNAse-free 1.5 mL eppendorf tube containing digestion buffer solution from the Recover Total Nucleic Acid Isolation kit (Ambion by Life Technologies, Austin, TX, USA), to proceed with RNA extraction.

### RNA Extraction from plasma samples

Total RNA was isolated from plasma using miRNeasy Serum/Plasma Kit (Qiagen, Hilden, Germany) according to the manufacturer’s protocol. RNA samples were quantified using *NanoDrop 8000* (Thermo Fisher Scientific, Waltham, MA, USA).

### RNA Extraction from FFPE samples

RNA from FFPE samples was isolated using the *RecoverAll Total Nucleic Acid Isolation* (Ambion/Life Technologies, Carlsbad, CA, USA), following a previously reported protocol with modifications to improve RNA yield^23^. RNA samples were quantified using *NanoDrop 8000* (Thermo Fisher Scientific, Waltham, MA, USA).

### Quantification of miRNA expression

miRNA expression was quantified using the TaqMan® Human MicroRNA Array Card (TLDA) assay (card A v3.0) (Life Technologies, Foster City, CA, USA), as previously described^24^. We used the QuantStudio 12 K system (Life Technologies, Foster City, CA, USA). Global data normalization was performed in Expression Suite software (Life Technologies, Foster City, CA, USA) and miRNA expression profiles were determined using RQ Manager v.1.2 software (Life Technologies, Foster City, CA, USA).

### Analysis of microRNA expression data

Data were extracted from the QuantStudio™ 12 K Flex equipment using ExpressionSuite Software v1.0.3 for Microsoft® Windows®, and analyzed using the ΔΔCT method^25,26^. This platform allows relative gene expression quantification through a large number of genes as well as Fold Change (FC) calculation. Correlation analysis between miRNA expression in plasma and rectal tissue samples as well as among tissues derived from the distinct intestinal layers was performed and visualized using InterractiVenn^27^ and evaluated by Spearman’s test. Categorical variables were expressed by the frequency and respective percentages. Continuous numerical variables were expressed as mean ± standard deviation for parametric data and as median (minimum/maximum) for nonparametric data. The significance level was 5%. Analyses were performed using SPSS software (IBM SPSS Statistics for Windows, Version 22.0. NY, USA).

### miRNA target prediction and pathway identification

miRNAs identified as deregulated in rectal biopsy tissues and plasma were subjected to target prediction analysis using microRNA Data Integration Portal (http://ophid.utoronto.ca/mirDIP/)^28^, a bioinformatic tool that integrates sequence information and prediction data from multiple sources. This analysis was performed using as criteria for target selection, results with “very high” (top 1%) scores for interaction probability. Statistically enriched pathways of genes regulated by miRNAs were identified using ToppGene Suite (https://toppgene.cchmc.org/)^29^.

## Results

Fifty IND-B patients participated in the study. Thirty-five patients (70%) were male. Median age of patients at the time of diagnosis was 3 years (0–15 years). The average time between diagnosis and blood samples collection was 9.34 ± 3.34 years. Mean age of IND-B patients at the time of blood sample collection was 12.32 ± 5.07 years. Median age of volunteers at time of blood sample collection was 2.5 years (0–14 years). Seven volunteers were female and 3 were male.

### Identification of plasma microRNA expression profile

We identified 32 miRNAs statistically significantly deregulated (fold change - FC ≥ 2 and *p* ≤ 0.05) in patients with IND-B compared to heathy controls, with 14 miRNAs having increased expression and 18 miRNAs having decreased expression (Table 1).

**Table 1:** miRNAs with altered expression in plasma of IND-B patients.

Over-expressed			Under-expressed		
miRNA	FC	*p*	miRNA	FC	*p*
let-7a-5p	17.409	0.015	miR-195-5p	0.410	0.044
miR-342-5p	6.572	0.034	miR-106b-5p	0.371	0.028
miR-98-5p	6.672	0.000	miR-337-5p	0.367	0.029
miR-196b-5p	5.159	0.031	miR-429	0.266	0.047
let-7e-5p	5.177	0.021	miR-101-3p	0.254	0.047
miR-758-3p	4.827	0.017	miR-590-5p	0.302	0.025
miR-361-5p	4.769	0.005	miR-362-3p	0.311	0.013
miR-125a-5p	4.009	0.037	miR-19a-3p	0.281	0.011
miR-155-5p	3.831	0.034	miR-19b-3p	0.252	0.009
let-7d-5p	4.113	0.014	miR-636	0.361	0.005
miR-539-5p	3.885	0.005	miR-889-3p	0.325	0.004
miR-423-5p	2.672	0.021	miR-210-3p	0.332	0.003
miR-99b-5p	2.210	0.036	miR-193b-3p	0.252	0.005
miR-491-5p	2.195	0.023	miR-214-3p	0.193	0.046
			miR-500a-5p	0.207	0.016
			miR-885-5p	0.221	0.007
			miR-483-5p	0.218	0.004
			miR-451a	0.186	0.004
			miR-660-5p	0.154	0.032

FC: fold change.

### Identification of microRNA expression profile in FFPE rectal tissue samples

We aimed to identify miRNA expression in the different layers of the intestinal wall (mucosa, submucosa and muscular layer). The mucosal layer was set as the tissue in which there was no nerve plexus of the enteric nervous system, since ganglion cells of the nerve plexus, which are mostly affected in IND-B patients, are not located in this layer, but in the submucosa (Meissner’s Plexus) and in the muscular layer (Auerbach’s Plexus).

### miRNAs deregulated in the submucosa layer

11 miRNAs were statistically significantly deregulated (3 over-expressed and 8 under-expressed) in submucosa compared to the mucosal layer (FC ≥ 2 and *p* ≤ 0.05) (Table 2).

**Table 2:** miRNAs with altered expression in the submucosa intestinal layer.

miRNA	FC	*p*
**Over-expressed**		
miR-146b-5p	4.683	0.039
miR-146a-5p	4.184	0.040
miR-127-3p	2.278	0.009
**Under-expressed**		
miR-451a	0.401	0.016
miR-99a-5p	0.380	0.023
miR-130a-3p	0.291	0.046
miR-374b-5p	0.272	0.041
miR-100-5p	0.197	0.038
miR-365a-3p	0.095	0.027
miR-145-5p	0.136	0.010
miR-133b	0.159	0.001

FC: fold change.

### miRNAs deregulated in submucosa plus muscular layers

29 miRNAs were statistically significantly deregulated (3 over-expressed and 26 under-expressed) (FC ≥ 2 and *p* ≤ 0.05) in the submucosa + muscular layers compared to the mucosal layer (Table 3).

**Table 3:** miRNAs with altered expression in submucosa plus muscular layers.

miRNA	FC	*p*
**Over-expressed**		
miR-134-5p	3.953	0.040
miR-146a-5p	4.577	0.026
miR-146b-5p	4.547	0.003
**Under-expressed**		
miR-20b-5p	0.470	0.018
miR-10a-5p	0.399	0.022
miR-744-5p	0.349	0.022
miR-24-3p	0.347	0.029
miR-99b-5p	0.247	0.030
miR-139-5p	0.229	0.044
miR-628-5p	0.256	0.027
miR-100-5p	0.262	0.021
miR-24-3p	0.347	0.029
miR-99b-5p	0.247	0.030
miR-139-5p	0.229	0.044
miR-628-5p	0.256	0.027
miR-100-5p	0.262	0.021
miR-15b-5p	0.299	0.019
miR-487b-3p	0.354	0.016
miR-28-3p	0.333	0.011
miR-27b-3p	0.411	0.009
miR-132-3p	0.293	0.008
miR-374b-5p	0.309	0.007
miR-125a-5p	0.277	0.004
miR-451a	0.361	0.002
miR-99a-5p	0.346	0.002
miR-130a-3p	0.224	0.006
miR-133b	0.175	0.017
miR-23b-3p	0.136	0.016
miR-28-5p	0.147	0.006
miR-125b-5p	0.175	0.003
miR-143-3p	0.159	0.003
miR-1-3p	0.072	0.009
miR-145-5p	0.095	0.001
miR-365a-3p	0.111	0.001

FC: fold change.

### A subset of miRNAs is commonly deregulated in submucosa and submucosa plus muscular layers

**Figure 2:**
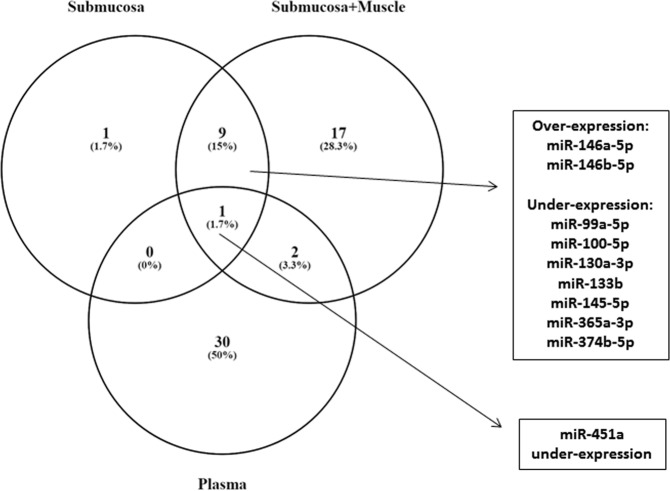
Venn diagram illustrating the number of altered miRNAs in FFPE rectal biopsies: submucosal layer and submucosal plus muscular layer and plasma samples. Commonly over- or under-expressed miRNAs are shown, as indicated by the arrows and respective boxes.

miR-451 was commonly deregulated (under-expressed) in plasma and biopsy samples from the same patients (Fig. 2). There was a statistically significant correlation between the expression of a subset of 10 miRNAs that were simultaneously deregulated in submucosa layer and in submucosa + muscular layer (r = 0.9394; *p* < 0.001). There was no statistically significant correlation between the expression quantification of the 3 miRNAs that were simultaneously deregulated in submucosa layer and in plasma (r = 0.50; *p* = 0.667).

### miRNA target genes are related to pathways of neuronal control

**Figure 3:**
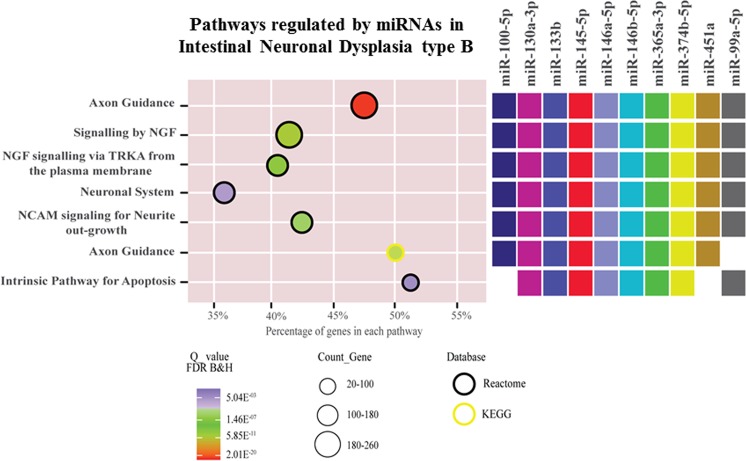
Enriched pathways. miRNAs regulate a wide range of genes with roles in mechanisms related to intestinal neuronal control.

miRNA target prediction analysis using mirDIP showed 8,616 interactions (Supplemental Table 1). Of these, 5,164 exclusive genes were involved in 295 significantly (p ≤ 0.01) enriched pathways (Supplemental Table 2). Of these, the most enriched pathways based on statistical significance as well as the number of involved genes (>50% enrichment) were axon guidance, nerve growth factor (NGF) signaling, NCAM signalling for neurite out-growth, neuronal system and apoptosis (Fig. 3) (Supplemental Table 3).

## Discussion

IND-B is a rare disease that remains surrounded by several controversies ranging from its definition as a real disease to the different criteria for its diagnosis and treatment options^2,7,30^. Faced with all these uncertainties, physicians are routinely challenged about what to do with the results of rectal biopsies that reveal submucosal nerve plexus hyperplasia, leading to the diagnosis of IND-B in patients with severe constipation or intestinal obstruction^7^. Thus, it is necessary to conduct studies focused on the investigation of the origin and pathophysiology of this disease.

One strength of this study is the microdissection performed in archived FFPE rectal tissues, exploring the different layers of the intestinal wall. Thus, it was possible to analyse the expression of miRNAs at the site where the histopathological changes of the IND-B are found. In addition, miRNA expression analysis was performed using a platform with high sensitivity and specificity^23^. These combined strategies led to mapping miRNAs that regulate genes that plays important functions, potentially related to the development of the disease. Among our study limitations, we included patients from a single center, with a limited sample size. Our sample size is justified due to the rarity of IND-B, with an estimated incidence of approximately 1 per 7,500 newborns^31^. Although, the 50 patients included in our study represent the largest case series of IND-B patients with associated molecular data, to date. In addition, our study does not include rectal biopsy tissues from healthy controls. Obtaining rectal biopsies from healthy, asymptomatic children, to be used as controls, is a great challenge, due to the potential complications of an invasive procedure, which is also not ethically accepted. In other studies^14,32–34^, rectal tissue samples used as controls were obtained from patients with intestinal congenital malformations, such as anorectal anomalies or other intestinal dysganglionosis, which cannot be considered healthy controls. Limitations related to the lack of healthy controls in IND-B studies are widely discussed in the literature^4,14,30^. Due to this reason, our analysis strategy was to compare miRNA expression levels in intestinal submucosa and submucosa plus muscular layers against the epithelial mucosal layer. This strategy is valid since the ganglion cells of the nerve plexus, which are affected in IND-B, are not present in the mucosal layer.

miRNAs are attractive for the molecular characterization of human pathologies, as well as biomarker development, since these molecules are abundant, stable and can be detected both in tissue biopsies as well as circulating in body fluids^16,18–21,24^. Of note, our study used needle-microdissected biopsy tissues and profiled miRNA expression associated with the different layers of the intestinal wall. In fact, miRNAs play regulatory roles in the different cell compartments in the intestines^35^. This stringent criterion of sample selection and analysis allowed us to identify deregulated miRNAs specific to the submucosal intestinal layer, which contains neuronal cells, and may thus reflect IND-B pathogenesis. miRNAs that were deregulated in the submucosal layers from all IND-B patients were also detected as deregulated in pooled samples of submucosal plus muscular layers; this result indicate that this commonly deregulated subset may be involved in IND-B development.

miRNAs which exhibited increased expression (miR-146a, miR-146b) or decreased expression (miR-451, miR-99a, miR-130a, miR-374-5p, miR-100, miR-365, miR-145, miR-133b) simultaneously in the submucosa and in the association between the submucosa and muscular layers, may have activity related to neurons of the enteric nervous system and be potentially involved in the pathophysiology of IND-B. Interestingly, miR-146a was demonstrated to regulate inflammatory cytokines including *NOS2*, *TNFα*, *COX-2*, *IL-6* and *IL-8* in human glial cells^36^. *NO2* gene encodes a nitric oxide (NO) synthase with different functions in several tissues. NO signalling pathways contribute to a number of physiologic functions in the gastrointestinal system, including motility and vascular regulation, as well as disease processes related to these functions^37^. miRNAs including miR-146a have been shown as a potential regulators of bovine gastrointestinal tract, with roles in tissue cell proliferation and differentiation^38^. Notably, miR-146a and miR-146b have ~70% homology with conserved sequences in human and bovine genomes, according to data retrieved from miRviewer, a computational tool that allows comparison between miRNAs in multiple species^39^. In addition, miR-146 was described as a potential player in neonatal innate immunity^40^ and in proliferation and differentiation of mouse neural stem cells^41^. Considering the literature evidence on the modulatory roles of miR-146 in inflammation, gastrointestinal development, motility and cell proliferation, deregulated levels of miR-146a and miR-146b, as shown in our study, may be implicated in IND-B pathogenesis.

In IND-B patient plasma, we identified several over- or under-expressed miRNAs. miRNA let-7a showed high expression (17-fold) in plasma from patients with IND-B, compared to healthy controls. miRNA let-7 belongs to a family of miRNAs present in multiple genomic locations, and consists of 9 members^42,43^. Genes such as *cyclin A2*, *CDC34*, *Aurora A* and *B kinases* (*STK6* and *STK12*), *E2F5*, and *CDK8* were characterized as responsive to changes in miRNA let-7a levels. These genes are related to the regulation of the cell cycle, involving mechanisms of apoptosis and cellular proliferation^44^, which are biological processes that could explain the hyperplasia of nerve plexuses of the enteric nervous system that occurs in IND-B. Furthermore, miR let-7 suppresses the differentiation of human embryonic stem cell derived neural progenitor cells and influences self-renewal of neural stem cells^43,44^. Over-expression of let-7a influences neural stem cells proliferation and differentiation^43,45^. However, the mechanisms of this interaction have not been elucidated yet^43^.

Interestingly, miR-451 was consistently under-expressed in all layers of the intestinal wall and detected as under-expressed in patient plasma; these data suggest a possible role for miR-451 in the pathophysiology of IND-B. miR-451 has been extensively studied in recent years and is related to cell proliferation and apoptosis, in inflammatory processes such as rheumatoid arthritis and in colorectal cancer^46–49^. Cheung Ng *et al*. (2015) observed increased expression of miR-451 in small bowel samples from patients with necrotizing enterocolitis (22-fold) and spontaneous intestinal perforation (8-fold) compared to small bowel samples of children without acute inflammatory processes, but who had been subjected to surgical treatment due to congenital malformations, such as intestinal atresia and meconium ileus^50^. One must observe that increased miR-451 expression was detected as compared to patients with congenital intestinal malformations, such as IND-B. Therefore, its expression may be actually reduced in IND-B patients, in agreement with our findings. There is also evidence that miR-451 may regulate GNA11 protein synthesis, which plays an important role in the modulation of transmembrane systems, including intestinal muscle contraction^50^. In addition, Alural *et al*. (2014) observed that downregulation of miR-451 can lead to a neuroprotective and an anti-apoptotic effect in an *in vitro* model of SH-SY5Y neuron-like cells^51^.

Our study provided information on miRNA target genes involved in the regulation of several pathways that may be related to IND-B pathogenesis (literature experimental data, Supplemental Table 3). Among several genes regulated by miRNAs, which play roles in the identified pathways, *RET*, *GNDF* and *RAS* have an important role in differentiation of neural crest cells through signal transduction mechanisms during enteric nervous system development^52^. In our study, most enriched pathways identified were axon guidance, nerve growth factor (NGF) signalling, neural cell adhesion molecule (NCAM) signalling for neurite out-growth, neuronal system and apoptosis. Axon guidance regulates synaptogenesis, progenitor axon cell dynamics, cell migration and neural circuit formation, through different mechanisms^53^. NGF is a neurotrophic factor that mediates biological mechanisms in neuronal cells through nerve regeneration, germination of nerves, protection of damaged neurons and promotion of inflammatory response and revascularization of recurrent nerves. Recently, NGF signaling has been associated with diarrhea-predominant irritable bowel syndrome. NGF signaling likely exerts its functions including other signaling, regulatory molecules and complex signaling networks/pathways^54^. Additionally, NCAM signalling has a wide range of biological effects, including formation and maintenance of the nervous system, axonal growth, survival and synaptic plasticity in neurons^55^. Considering these experimental evidence from the literature, together with a large fraction of genes in our data involved in significantly enriched pathways (40–50%, Supplemental Table 3), we suggest that miRNA-gene interaction networks identified here may contribute to IND-B pathogenesis.

The definition of IND-B as a genetic disease has been raised since the description of monozygotic twins with IND–B and by the report of similar symptoms and histological findings of IND-B in several members of a family, through multiple generations^56^. Although mutations in genes related to the etiology of HD, such as RET, glial cell line-derived neutrophic factor (GDNF) have not been identified in IND-B, some studies attempted to link these two conditions in the same molecular pathways^32,33,57–60^. To date, only a few combinations of polymorphisms in the RET proto-oncogene was described in IND-B patients^34^.

The experimental studies trying to elucidate the pathogenesis of IND-B also demonstrate conflicting results. Although experiments with rodents have demonstrated the occurrence of megacolon and hyperplasia of the myenteric nerve plexus in homozygotic animals with NCX/Hox11L.1 gene deficiency, these changes were not demonstrated in humans with IND-B^10,11,57^. In addition, Holland-Cunz *et al*. (2003) demonstrated that mice presenting heterozygous deficiency in endothelin B receptor (EDNRB) showed histopathological alterations compatible with IND-B, but had no clinical symptoms of bowel obstruction^61^. These alterations were also not found in humans^32^.

We provided evidence that there are alterations in the expression of certain miRNAs in the rectal submucosa and muscular layer, which are the layers where normally the ganglion cells are located. Additionally, we showed miRNA expression changes in plasma of patients with IND-B, compared with healthy individuals. Biologically enriched pathways regulated by the identified miRNAs may play a role in IND-B disease pathogenesis, due to the activity related to the neurons of the enteric nervous system. These findings reinforce the theory that molecular determinants may be drivers of IND-B pathogenesis. In this context, IND-B should be understood as a real disease, characterized by specific histopathological and molecular changes. Our findings on deregulated miRNA expression and miRNA-gene interactions and enriched pathways contribute with novel and valuable information on disease pathogenesis. Our data may be the basis for future studies focusing on miRNAs as potential clinical biomarkers to improve disease diagnosis.

### Informed consent

Informed consent was obtained from all individual participants included in the study.

### Data sharing statement

Original, raw data is available upon request to the corresponding author: pedro.lourencao@unesp.br

## Supplementary information

Supplementary Tables
